# Characteristics of Pharmacists' Interventions Related to Proton-Pump Inhibitors in French Hospitals: An Observational Study

**DOI:** 10.1155/2022/9619699

**Published:** 2022-06-28

**Authors:** AL Yailian, E. Huet, B. Charpiat, O. Conort, M. Juste, R. Roubille, M. Bourdelin, J. Gravoulet, C. Mongaret, C. Vermorel, P. Bedouch, A. Janoly-Duménil

**Affiliations:** ^1^Pharmacy Department, Edouard Herriot Hospital, Hospices Civils de Lyon, Lyon, France; ^2^Université Claude Bernard Lyon 1, Université de Lyon, UR 4129 Parcours Santé Systémique, Lyon, France; ^3^Pharmacy Department, Dieppe Hospital, Dieppe, France; ^4^Pharmacy Department, Croix Rousse Hospital, Hospices Civils de Lyon, Lyon 69004, France; ^5^Université Grenoble Alpes, CNRS/TIMC UMR5525, F-38041, Grenoble, France; ^6^Working Group ‘Valorization of Pharmaceutical Interventions/Valorisation des Interventions Pharmaceutiques – Act-IP©' of the French Society for Clinical Pharmacy, Marseille, France; ^7^Clinical Pharmacy Department, Cochin Hospital, APHP Centre, Cochin, Université de Paris, Paris, France; ^8^Pharmacy Department, Auban Mouet Hospital, Epernay, France; ^9^Pharmacy Department, Lucien Hussel Hospital, Vienne, France; ^10^Pharmacy Department, Nord-Ouest Villefrance Hospital, Villefranche sur Saône, France; ^11^Regional Union of Healthcare Professionals Pharmacists of the Grand Est (URPS), Nancy 54000, France; ^12^Lorraine University, Faculty of Pharmacy, Nancy 54000, France; ^13^Pharmacy Department, Reims University Hospital, Reims, France; ^14^Reims Champagne Ardennes, Faculty of Pharmacy, Reims, France; ^15^Pharmacy Department, Grenoble Alpes University Hospital, Grenoble 38043, France

## Abstract

**Methods:**

The study was based on a retrospective analysis of pharmacist interventions for DRPs detected during the medication order review and documented into the French Act-IP© database over a 12-year period. DRPs and PIs were analyzed, and independent factors of physician acceptance were assessed via multiple logistic regression.

**Results:**

Out of the 620,620 PIs registered, 29,694 targeted a PPI (4.8%). PPI's DRPs were mostly related to the prescription of a “drug not available at the hospital” (26.1%) and a “drug use without indication” (18.3%); PIs were mostly “drug switch” (35.9%) and “drug discontinuation” (26.1%). In all, 18,919 PIs were accepted by physicians (63.7%). Acceptance was significantly associated with patient age: less accepted for the 18–75 years group (OR = 0.59, 95 CI [0.46–0.76]), and the >75 years group (OR = 0.57, 95 CI [0.44–0.73]) vs. <18 years group; for the type of DRP, “drug use without indication” was the less accepted (OR = 0.73, 95 CI [0.63–0.85]); for the type of PI, “dose adjustment” was the less accepted (OR = 0.32, 95 CI [0.23–0.45]).

**Conclusion:**

Pharmacists contribute to preventing DRPs associated with PPI prescriptions during the medication order review process. Moreover, they often detect PPIs used without indication and they propose drug discontinuation, which contributes to the PPI deprescribing process. PIs should be further developed in the future to reduce PPI overprescription.

## 1. Introduction

The introduction of proton-pump inhibitors (PPIs) in clinical practice has greatly improved the therapeutic management of acid-related gastrointestinal diseases in light of their efficacy and safety [1, 2]. The main validated indications for PPIs are gastroesophageal reflux disease management, eradication of *Helicobacter pylori* (H.p.) infection in combination with antibiotics, treatment of (H.p.)-negative peptic ulcer disease, treatment and prophylaxis of NSAID-associated gastric ulcers, and management of gastric acid hypersecretory states [1]. Despite their efficacy, long-term use of PPIs has been linked to several adverse effects [1, 3–5].

While the use of PPIs should be limited to well-defined clinical indications, they currently represent one of the most prescribed drugs worldwide [6]. Outpatient prescription rates are very high, with more than 60% of PPIs prescribed without a valid indication [7]. Even if inappropriate PPI prescription is common in primary care [8], it is often initiated during patient hospitalization [4, 8] and continued post-discharge by primary care physicians, often indefinitely [4, 9]. Some of the PPIs are appropriately prescribed (into hospital and ambulatory care) but the appropriateness becomes inappropriate over time. Another aspect of the overprescription is the long-term use of high-dose PPIs after acute prescription particularly after discharge. Overprescribing PPIs has become the norm worldwide [10] and represents a global health challenge leading to economic consequences [2].

Rare collaborative studies were conducted to improve PPI use in primary and secondary care. In France [11–13] and elsewhere [14–16], these studies were all monocenter.

To date, no large study has focused on PPI-related pharmacists' interventions (PIs) formulated during the medication order review. Consequently, at national level, we do not know how hospital pharmacists are involved in inappropriate PPI prescriptions.

In 2006, the French Society of Clinical Pharmacy (SFPC) developed the Act-IP©. This is a web-based observatory of PIs, enabling hospital pharmacists to document and analyze PIs undertaken during the medication order review process when a DRP was detected [17]. PIs were recorded based on the coding system described previously [18]: patients' demographic characteristics, drugs involved, wards, DRP description, pharmacists' recommendations, and whether or not the recommendations were accepted by the physicians. PI collection on this web-based tool constitutes an observatory of clinical pharmacy practices in French hospitals.

Thus, the study aimed at assessing pharmacist's contribution to PPI deprescribing.

The primary objective was to analyze DRPs associated with PPI prescriptions detected during the medication order review process in French hospitals and associated pharmacists' interventions (PIs). The second objective was to identify factors associated with physicians' acceptance of PIs.

## 2. Methods

### 2.1. Study Design

This was a French observational study based on prospectively documented PIs issued from medication order review in the Act-IP© observatory over a 12-year period from January 1, 2007, to December 31, 2019.

Medication order review modalities depended on the care setting and could be performed in wards or in a central pharmacy. Pharmacists had access to the complete drug order and laboratory results; they could be present in the wards for medication order validation to obtain more information on drug therapy and clinical aspects, for example, by participating in the medical rounds. In this study, the clinical pharmacist often was a senior pharmacist or resident pharmacist.

For each order, the pharmacist had to analyze the suitability of treatment by referring to medication orders and the patient's medical record when available. Thus, pharmacists have the opportunities to detect DRPs and implement pharmacists' interventions (PIs), defined as any action initiated by a pharmacist directly resulting in a physician adjusting the patient's treatment or its management [19]. Then, pharmacists registered on the Act‐IP© website [20] have a unique user login and password and can log on from any location to document and analyze their PIs.

### 2.2. Pharmacists' Intervention Data

Among all PIs recorded over the study period in Act-IP©, PPI-related interventions (omeprazole, pantoprazole, lansoprazole, rabeprazole, and esomeprazole) were extracted from the Act-IP© following the Anatomical Therapeutic Chemical (ATC) classification. For each PI, the date, hospital department (psychiatry care, rehabilitation, short-term acute care, and long-term care), healthcare institution (university teaching hospital, general hospital, psychiatric hospital, and other healthcare institutions), patient characteristics (age and gender), DRP description, specific intervention, and physician acceptance were completed. A PI was considered accepted by physicians when they took it into account for the treatment (i.e., prescription modification or clinical/biological follow-up).

### 2.3. Data Analysis

PPI-related problem data were extracted from the Act-IP©, containing 620,620 PIs recorded over the study period. Statistical analyses were performed using Stata®, version 15.0 (StataCorp, College Station, TX, USA).

#### 2.3.1. DRP and PI Characteristics

Descriptive statistics were used for characterizing DRPs and PIs. DRP evolution was analyzed over the study period. DRP ratio according to patient age (<18; [18–75]; ≥75 years old) and hospital department along with PI ratio according to patient age and hospital department were compared using the Pearson's chi-squared test. Statistical significance was considered when *p* value was <0.05.

### 2.4. Physicians' Acceptance and Factors of Acceptance

For the purpose of this study, PIs assessed as refused were compared with accepted PIs. PIs coded as not assessable were removed from the analysis. The following variables were tested via univariate analysis to detect a possible correlation with PI acceptance: healthcare institution, patient age (<18; [18–75]; ≥75 years old) and gender, hospital department, DRP, PI, and year the PI was documented. Variables significantly correlated with PI acceptance (*P* < 0.2) via the univariate analysis were included in the multivariable model. The multivariate analysis was performed by logistic regression. Results were presented as odds ratios (ORs) and 95% confidence intervals (CIs).

## 3. Results

Out of the 620,620 PIs registered over the study period, 29,694 PIs (4.8%) involved a PPI. The percentage of DRPs associated with PPI prescriptions was stable over time (Supplementary Appendix A). 29,694 PIs were issued at 328 hospitals. In total, 659 PIs (2.2%) pertained to a pediatric patient (<18 years), 15,665 (52.8%) to [18–75] years adult patient, and 13,326 PIs (44.9%) involved patient >75 years. The M/F gender ratio was 1.0. The hospital department with the most DRPs was short-term acute care (73.5%) (Table 1).

### 3.1. DRP Characteristics

DRP characteristics are summarized in Table 1. The most common DRP identified by pharmacists was “prescription of a drug not available at the hospital pharmacy” (*n* = 7,750; 26.1%), followed by “drug use without indication” (*n* = 5,544; 18.3%) and “improper administration” (*n* = 4,852; 16.3%). Comparatively, among the 620,620 DRPs registered over the study period, “prescription of a drug not available at the hospital” represented 60,799 DRPs (9.8%), “drug use without indication” represented 55,081 DRPs (8.9%), and “improper administration” represented 96,515 DRPs (15.6%). There was a significant difference in DRP types and year the PI was recorded (Supplementary Appendix B): there was an increase of “drug use without indication” (15.9 to 23.5%) and “untreated indication” (0.9 to 10.1%) DRPs, between 2007 and 2019. There was a decrease of “improper administration” (23.4 to 11.1%) during this period. There was an increase of “drug interaction” DRP detection in 2009 and 2010 followed by a decrease afterwards. In 2010, this DRP was 3 times higher than that in 2008.

DRPs were significantly different according to patient age (*p* < 0.01) (Table 2). The most frequent in the pediatric population (<18 years) was “improper administration” (*n* = 232; 35.2%). In adults (>18 years), the most common was “prescription of a drug not available at the hospital pharmacy” (*n* = 4,395; 28.1%).

DRPs were significantly different according to the hospital department (*p* < 0.01) (Table 3). The DRPs “drug use without indication” and “supratherapeutic dose” were more often identified for patients in long-term care (*n* = 828; 31.7% and *n* = 373; 14.3%) or rehabilitation care (*n* = 939; 21.3% and *n* = 829; 18.8%) than in short-term acute care (*n* = 3,430; 15.7% and *n* = 3,455; 15.8%). “Improper administration” was more frequently reported in short-term acute care (17.3%) than in other departments (<15%). “Prescription of a drug not available at the hospital” was more often detected in short-term acute care (29.2%) or psychiatry (36.9%) than in rehabilitation care (18.6%) or long-term care (14.2%).

#### 3.1.1. Nature of Pharmacists' Interventions

Main PIs proposed were “drug switch” (*n* = 10,672; 35.9%), followed by “drug discontinuation” (*n* = 7,751; 26.1%).

PIs were significantly different according to patient age (*p* < 0.01) (Table 2). In pediatrics (<18 years), the most frequent PIs were “drug switch” (*n* = 180; 27.3%) and “administration modality optimization” (*n* = 167; 25.3%). In adults ([18–75] and ≥75 years, respectively), most frequent PIs were “drug switch” (*n* = 5,684; 36.3% and *n* = 4,788; 35.9%) and “drug discontinuation” (*n* = 4,082; 26.1% and *n* = 3,557; 26.7%).

PIs were significantly different according to the hospital department (*p* < 0.01) (Table 3) with higher rate of “drug discontinuation” in rehabilitation (*n* = 1,360; 30.8%), psychiatry (*n* = 117; 29.9%), and long-term care (*n* = 961; 36.8%) than in short-term acute care (*n* = 5,127; 23.5%) and higher rate of “drug switch” in short-term acute care (*n* = 8,537; 39.1%) and in psychiatry (*n* = 160; 40.8%) than in other departments (*n* = 1,268; 28.7% rehabilitation and *n* = 590; 22.6% long-term care). “Dose adjustment” was more frequent in rehabilitation (*n* = 1,016; 23.2%) and long-term care (*n* = 641; 24.5%) than in short-term acute care (*n* = 3,501; 16.0%) and in psychiatry (*n* = 67; 17.1%). “Change of administration route” (from intravenous to oral administration) was found more in short-term acute care than in other hospital departments.

#### 3.1.2. Physician Acceptance

Out of the 29,694 PIs, 6,006 were coded as not assessable for acceptance and removed from the analysis. Among the 23,688 remaining PIs, 18,919 (79.9%) were accepted by physicians. The acceptance rate was 87.1% (507/582) in the pediatric population and 79.5% (18377/23106) in the adult population. The rate of acceptance was significantly associated to the nature of PI (Table 4): a better acceptance was observed for the PI “addition of a new drug” (83.8%), and a lower acceptance was observed with “drug discontinuation” (73.1%) and “dose adjustment” (70.3%).

Results of the univariate and multivariate analysis are presented in Table 5.

The univariate analysis identified 6 variables that were related to PI acceptance: patient age, healthcare institution, hospital department, DRPs, PIs, and year the PIs were documented. Patient age, healthcare institution, hospital department, DRPs, and PIs remained statistically significant in the multivariate analysis. Concerning patient's age, PIs were less accepted for the 18–75 (OR = 0.59, CI 95 [0.46–0.76]) and >75 (OR = 0.57, CI 95 [0.44–0.73]) age groups vs. < 18 age group. For DRP, “drug use without indication” was the least accepted (OR = 0.73, CI [0.63–0.85]). Regarding PI, “dose adjustment” was the least accepted (OR = 0.32, CI [0.23–0.45]).

## 4. Discussion

This analysis of the Act-IP© national observatory database reports the involvement of French hospital pharmacists in the management of DRPs associated with PPI prescriptions with 29,694 PIs registered between 2007 and 2019. This is the first large-scale study focusing on PPI-related PIs formulated during the medication order review process.

Comparing all DRPs (Table 1) from the Act-IP© database, the profile of DRPs associated with PPI prescriptions is different, particularly there were a lot more “drug use without indication.” Furthermore, despite the fact that the rate of PPI-related PIs has remained relatively stable over time, there has been an increase in DRPs between 2007 and 2019 for “use of drugs without indication,” highlighting the growing contribution of French hospital pharmacists in the process of PPI deprescribing (Supplementary Materials Appendix B).

Another form of inappropriate drug use is the choice of intravenous route instead of oral administration [21]. PPIs are too often prescribed intravenously vs. the cost-effective enteral or oral route, when patients can take the medicines orally [11]. In the present study and in line with data from the literature, about 16.3% of the PPI-related PIs were associated to improper administration mode. Nevertheless, we observed a decrease for this DRP over time (Supplementary Materials Appendix B), suggesting improved prescribing practices.

Concerning the increase of the “untreated indication” DRP in the last years of our study (Supplementary Materials Appendix B), it can be surprising given the PPI overprescribing issue. It is probably related to the development of a reconciliation medication process at hospital admission, allowing to detect omitted medications [22]. As PPIs are often prescribed before admission (medication prescribed for regular use), they can be omitted on the patient's chart and fall under “untreated indication” category during the medication order review.

### 4.1. Hospital Department Influences DRP Type

In long-term care, there were statistically more “drug use without indication” (31.7%) DRPs than in short-term acute care (15.7%). Detecting this DRP is easier in long-term care than in short-term care. In addition, the use of drugs without an indication is more likely to occur in a long-term care, as the acute treatment should have been stopped in that setting, as opposed to an acute scenario where the intention is to use a PPI in the short term, but often without specifying a duration of treatment. “Drug use without indication” DRPs might be underestimated in short-term acute care as pharmacists could be less comfortable detecting it in this setting. “Deprescribing a PPI without indication” was more accepted by physicians in long-term care. In short-term care, physicians and patients might not be aware of PPI indications and treatment course. Thus, the treatment is rarely discontinued at the hospital. To change this, it might be valuable to increase communications with the primary care physician or promote the patient's involvement in therapeutic decisions [23, 24].

### 4.2. Patient Age Influences DRP Type

“Improper administration” was more frequent in <18 years (35.2%) than in adults (15.5% in the 18–75 years group and 16.3% in the >75 years group). In this study, the “drug use without indication” rate was higher in the ≥75 years group (20.4%) than in other groups (11.2% in <18 years and 16.9% in 18–75 years). In the same way, observational studies reported the frequent overuse of PPIs in geriatrics (25).

The global acceptance rate in our study (79.9%) matched data from the literature, and physicians did not discriminate between PPIs or other drugs when it came to PI acceptance [25]. In the present study, the acceptance rate of PPI-related PIs was higher than in the previous study by Skalli et al. (69, 6%) [12]. The rate of “drug use without indication” DRP was higher in that study (24.4%) than in the present one (18.3%) and could partly explain this difference. In fact, physicians are more reluctant to deprescribe a drug than adding a new one, as underlined in our study, with a better acceptance rate for the “adding a new drug” PI (83.8%) than “drug discontinuation” (73.1%). Moreover, the “prescription of a drug not available at the hospital pharmacy” and subsequent PIs requesting drug substitution were more frequent in the present study (26.1% vs. 13% in the previous study). This PI is well accepted by physicians in the present study (88% acceptance rate). PI acceptance was associated to patient age, DRP, PI, institution, and department. Overall, physicians accepted more PIs in pediatrics than in adult populations, in accordance with previous studies [25]. Moreover, in regard to the “non-conformity to guidelines” DRP, physicians were more inclined to accept interventions on drug interaction, improper administration, and subtherapeutic or supratherapeutic dosage. Physicians appeared more reluctant to change their prescriptions for a “drug use without indication.” Compared to the well-accepted “addition of a new drug” PI, the acceptance rate was lower for all other PIs. Compared to psychiatry care, there is a better acceptance rate in short-term, long-term, and rehabilitation care. These data are similar to previous data from a study in a long-term psychiatric hospital reporting an overall 50% acceptance rate [26].

Despite the present study underlining an important increase over time of the “drug use without indication” DRP from 15.9 to 23.5%, according to PPI overuse in clinical practice [27], our results suggest that DRPs were poorly documented (4.8% of PIs) compared to the misuse rate of 19–86% reported in the literature [28]. Due to its time-consuming aspect, we can suppose that not all DRPs associated with PPI prescriptions are documented by pharmacists during the medication review process [25], and some pharmacists focus their PIs on others drugs. Moreover, some pharmacists specifically target their PPI-related PIs: in short-term care, pharmacists are more inclined to propose a change in dosage or administration route rather than stopping the treatment entirely. Nevertheless, in regard to PPI overprescribing, one of the most important safety concerns is to carefully evaluate PPI indication when initiating treatment and reconsider its indication for patients treated in the long term.

There is no increase in PPI acceptance over time (no correlation shown in multivariate analysis) while misuse and adverse effects and drug interactions are increasingly described in the scientific literature [1, 3–5, 8, 10, 27]. Data from the literature underlined that lack of time and perceiving PPIs as “harmless” affect physicians' decision making [29]. Physicians are sometimes unaware of PPI-related adverse effects. Ghosh et al. [30] reported that even though 60% of physicians interviewed reported concerns about PPI side effects, only 37% admitted to changing their practice based on these concerns [30].

Barriers to the deprescribing process include difficulty making the decision to stop medications (both from the clinician's and the patient's perspective), fear about stopping medications started by others, and insufficient knowledge about how to stop medications. Involving patients in the deprescribing process [31] and relying more on existing deprescribing algorithms are essential [32].

### 4.3. Limits

Our study bears some limitations. Firstly, it is likely that not all DRPs detected by pharmacists were documented in the database, due to lack of time or other reasons. Secondly, the number of total prescriptions analyzed was not documented in the Act-IP© database. It is therefore impossible to calculate a total number of PIs/total number of prescriptions ratio. Thirdly, PIs occurring during the medication order review are only one of the possible pharmaceutical actions to promote the proper use of PPIs. Other actions like prescription audits [11, 33], patient-pharmacist interview [31], medication reconciliation [22], and development of PPI deprescribing guidelines (algorithm) could improve prescription practices [13, 14, 32].

## 5. Conclusion

During the medication order review, hospital pharmacists detect a wide variety of PPI DRP. Moreover, they often detect PPIs used without indication and propose drug discontinuation, contributing to the PPI deprescribing process. Physicians appeared more reluctant to stop a PPI prescription or decrease dosage than adding PPIs to a treatment. Even if pharmacists might sometimes feel discouraged by the time-consuming work to be done in light of the overwhelming quantity of unjustified prescriptions, they must keep in mind the impact on the healthcare system with considerable clinical and economic consequences.

## Figures and Tables

**Table 1:** Characteristics of drug-related problems (DRPs).

Characteristics	PPI-related DRPs (%) (*n* = 29,694)	Total DRPs (*n* = 620,620)	PPI-related DRPs/total DRPs (%)
Patient			
Age (years)			
< 18	659 (2.2)	19,167 (3.1)	3.4
[18–75]	15,665 (52.8)	301,000 (48.5)	5.2
≥ 75	13,326 (44.9)	299, 570 (48.3)	4.4
Not specified	44 (0.15)	883 (0.1)	5.0

Gender			
Female	15,011 (50.5)	318,146 (51.3)	4.7
Male	14,683 (49.5)	302,474 (48.7)	4.9

Hospital department			
Short-term acute care	21,826 (73.5)	425,602 (68,6)	4.6
Long-term care	2,615 (8.6)	66,449 (10.7)	4.0
Rehabilitation	4,417 (14.9)	102,115 (16.5)	4.0
Psychiatry care	392 (1.3)	19,081 (3.1)	2.2
Not specified	444 (1.5)		

Drug-related problems			
Drug monitoring	119 (0.4)	21,470 (3.5)	0.6
Failure to receive drug	212 (0.7)	4,121 (0.7)°	5.1
Subtherapeutic dosage	1,020 (3.4)	51,666 (8.3)	2.0
Adverse drug reaction	366 (1.2)	13,100 (2.1)	2.8
Untreated indication	1,611(5.4)	51,430 (8.3)	3.1
Non-conformity to guidelines/contraindication	1,757 (5.9)	60,799 (9.8)	2.9
Drug interaction	1,777 (6.0)	42,498 (6.9)	4.2
Drug use without indication	5,440 (18.3)	55,081 (8.9)	9.9
Supratherapeutic dosage	4,790 (16.1)	135,257 (21,8)	3.5
Improper administration	4,852 (16.3)	96,516 (15.6)	5.0
Prescription of a drug not available at hospital	7,750 (26.1)	88,682 (14.3)	8.7

**Table 2:** Numbers and percentages of PPI-related problems and PPI-related pharmacist's interventions (PIs) according to age.

	*n* (%)			
	<18 years	[18–75] years	≥75 years	*P* value
PPI-related problems				
Non-conformity to guidelines/contraindication	60 (9.1)	999 (6.4)	698 (5.2)	<0.01^*a*^
Prescription of a drug not available at the hospital	84 (12.8)	4,395 (28.1)	3,256 (24.4)	
Drug monitoring	1 (0.2)	56 (0,4)	62 (0,5)	
Untreated indication	22 (3.3)	821 (5.2)	767 (5.8)	
Subtherapeutic dosage	29 (4.4)	499 (3.2)	492 (3.7)	
Supratherapeutic dosage	92 (13.7)	2,512 (16.0)	2,182 (16.4)	
Drug use without indication	74 (11.2)	2,649 (16.9)	2,712 (20.4)	
Drug interaction	35 (5.3)	980 (6.3)	755 (5.7)	
Adverse drug reaction	1 (0.2)	180 (1.2)	185 (1.4)	
Improper administration	232 (35.2)	2,431 (15.5)	2,177 (16.3))	
Failure to receive drug	29 (4.4)	143 (0.9)	40 (0.3)	

PPI-related PIs				
Addition of a new drug	34 (5.2)	873(5.6)	805 (6,0)	<0.01^*a*^
Drug discontinuation	108 (16.4)	4,082 (26.1)	3,557 (26.7)	
Drug switch	180 (27.3)	5,684 (36.3)	4,788 (35.9)	
Change of administration route	46 (7.0)	737 (4.7)	603 (4.5)	
Drug monitoring	5 (0.8)	335 (2.1)	320 (2.4)	
Administration modality optimization	167 (25.3)	1,136 (7.3)	888 (6.7)	
Dose adjustment	119 (18.1)	2,818 (18.0)	2,365 (17.8)	

^*a*^Numbers of “drug monitoring” and “failure to receive drug” DRPs were limited and were grouped for statistical analysis.

**Table 3:** Numbers and percentages of PPI-related problems and PPI-related pharmacist's interventions (PIs) according to the hospital department.

	*n* (%)				
	Psychiatry care	Rehabilitation	Short-term acute care	Long-term care	*P* value
PPI-related problems					
Non-conformity to guidelines/contraindication	19 (4.9)	357(8.1)	1,036 (4.8)	308 (11.8)	<0.01^*a*^
Prescription of a drug not available at the hospital	142 (36.9)	823 (18.6)	6,329 (29.2)	370 (14.2)	
Drug monitoring	0 (0.0)	10 (0.2)	77 (0.4)	32 (1.2)	
Untreated indication	14 (3.6)	312 (7.1)	1,168 (5.3)	89 (3.4)	
Subtherapeutic dosage	9 (2.3)	275 (6.2)	693 (3.1)	42 (1.6)	
Supratherapeutic dosage	68 (17.4)	829 (18.8)	3,455 (15.8)	373 (14.3)	
Drug use without indication	72 (18.3)	939 (21.3)	3,430 (15.7)	826(31.7)	
Drug interaction	27(6.9)	187 (4.2)	1,409 (6.5)	153 (5.9)	
Adverse drug reaction	1 (0.3)	46 (1.0))	293 (1.3)	25 (0.9)	
Improper administration	40 (10.7)	627(14.2)	3,779 (17.3)	322 (12.3)	
Failure to receive drug	0 (0.0)	12(0.3)	127 (0.6)	73 (2.8)	

PPI-related PIs					
Addition of a new drug	17(4.3)	330 (7.3)	1245 (5.7)	98 (3.8)	<0.01^*a*^
Drug discontinuation	117 (29.8)	1,360 (30.8)	5,127 (23.5)	961 (36.8.)	
Drug switch	160 (40.8)	1,268 (28.7)	8,537 (39.1)	590 (22.5)	
Change of administration route	1 (0.3)	151 (3.4)	1,196 (5.5)	38 (1.5)	
Drug monitoring	1 (0.3)	71 (1.6)	471 (2.2)	112 (4.2)	
Administration modality optimization	29 (7.4)	221 (5.0)	1749 (8.0)	175 (6.7)	
Dose adjustment	67 (17.1)	1,016 (23.2)	3,501 (16.0)	641 (24.5)	

^*a*^Numbers of “drug monitoring” and “failure to receive drug” DRPs were limited and were grouped for statistical analysis.

**Table 4:** Characteristics of pharmacists' interventions (PIs)a and physician acceptance.

Nature of intervention	PI related to PPIs		Acceptance		*P* value
	*n*	%	*n*	%	
Drug choice	16,140	68.0	13,237	82.0	<0.01^*a*^
Addition of a new drug	1,482	6.25	1,242	83.8	
Drug discontinuation	6,048	25.49	4,420,	73.1	
Drug switch	8,610	36.29	7,575	88.0	
**Dose adjustment**	**4,338**	**18.28**	**3,050**	**70.3**	
**Optimization of administration**	**2,823**	**11.90**	**2,297**	**81.4**	
Change of administration route	1,094	4.61	873	79.8	
Administration modality optimization	1,729	7.29	1,424	82.4	
**Drug monitoring**	**426**	**1.80**	**335**	**78.6**	
**Total**	**23,727**	—	**18,919**	**79.7**	

^*a*^For all categories.

**Table 5:** Association of different variables with pharmacist's intervention (PI) acceptance: results of the univariate and multivariate analysis.

Variable	Univariate model		Multivariate model		
	*χ* ^2^	*P value*	OR	95% CI	*P value*
Healthcare institution	30.47	<0.01	—	—	—
University hospital			1.00		
General hospital			0.82	0.77–0.88	≤0.001
Psychiatric hospital			1.71	0.78–3.74	0.18
Others			2.33	1.66–3.24	≤0.001

Patient age	31.55	<0.01			
< 18			1.00	—	—
[18–75]			0.59	0.46–0.76	≤0.001
≥ 75			0.57	0.44–0.73	≤0.001

Patient gender	0.004	>0.20	—	—	—

Hospital department	31.84	<0.01	—	—	NS
Psychiatry care			1.00	—	—
Short-term acute care			2.35	1.12–4.93	0.02
Long-term care			2.29	1.09–4.81	0.03
Rehabilitation			2.67	1.27–5.60	0.01

Drug-related problem	1,100.00	<0.01			
Non-conformity to guidelines/contraindication			1.00	—	—
Prescription of a drug not available at the hospital			3.61	3.01–4.34	≤0.001
Drug monitoring			0.65	0.39–1.09	0.11
Untreated indication			1.03	0.74–1.45	0.85
Subtherapeutic dosage			2.04	1.65–2.51	≤0.001
Supratherapeutic dosage			1.67	1.44–1.93	≤0.001
Drug use without indication			0.73	0.63–0.85	≤0.001
Drug interaction			1.20	0.99–1.44	0.07
Adverse drug reaction			1.00	0.73–1.37	0.98
Improper administration			1.55	1.29–1.85	≤0.001
Failure to receive drug			11.6	4.71–28.61	≤0.001

Pharmacist intervention	789 29	<0.01			
Addition of a new drug			1.00	—	—
Drug discontinuation			0.59	0.43–0.82	≤0.001
Drug switch			0.61	0.44–0.86	≤0.001
Change of administration route			0.53	0.36–0.76	≤0.001
Drug monitoring			0.72	0.47–1.09	0.12
Administration modality optimization			0.54	0.38–0.77	≤0.001
Dose adjustment			0.32	0.23–0.45	≤0.001

Year the PI was documented	45.40	<0.01	—	—	NS

## Data Availability

The data supporting the results were issued from the French national observatory Act-IP. Act-IP was created with the objectives to create a documentation system that is freely accessible to any pharmacist, through the Société Française de Pharmacie Clinique website (https://www.actip.sfpc.eu/actip/index/ficheip/), and pool the data recorded by all pharmacists to conduct epidemiological studies concerning DRPs detected by pharmacists. The pooling of PIs constitutes an observatory of clinical pharmacy practices, called the “Act-IP Observatory.”

## Additional Points

What is already known about this topic? Overprescribing proton-pump inhibitors (PPIs) has become the norm worldwide and represents a global health issue with tangible clinical and economic consequences. At national level, we do not know how pharmacists are involved in this issue. To date, no large study has focused on PPI-related pharmacists' interventions (PIs) formulated during the medication order review. What does this article add? Pharmacists contribute to preventing DRPs associated with PPI prescriptions during the medication order review process. They often detect PPIs used without indication and they propose drug discontinuation, which contribute to the PPI deprescribing process.

## Supplementary Materials

Supplementary Materials PPI-related DRPs among total DRPs according to year are presented in Appendix A. Nature of PPI-related DRPs according to year is presented in Appendix B.

## References

[1] Savarino V., Dulbecco P., de Bortoli N., Ottonello A., Savarino E. (2017). The appropriate use of proton pump inhibitors (PPIs): need for a reappraisal. *European Journal of Internal Medicine*.

[2] Forgacs I., Loganayagam A. (2008). Overprescribing proton pump inhibitors. *BMJ*.

[3] Bourne C., Charpiat B., Charhon N. (2013). Effets indésirables émergents des inhibiteurs de la pompe à protons. *La Presse Médicale*.

[4] Scarpignato C., Gatta L., Gatta A., Zullo A., Blandizzi C. (2016). Effective and safe proton pump inhibitor therapy in acid-related diseases - a position paper addressing benefits and potential harms of acid suppression. *BMC Medicine*.

[5] Salvo E. M., Ferko N. C., Cash S. B., Gonzalez A., Kahrilas P. J. (2021). Umbrella review of 42 systematic reviews with meta‐analyses: the safety of proton pump inhibitors. *Alimentary Pharmacology & Therapeutics*.

[6] Heidelbaugh J. J., Goldberg K. L., Inadomi J. M. (2009). Overutilization of proton pump inhibitors: a review of cost-effectiveness and risk in PPI. *American Journal of Gastroenterology*.

[7] Thomas L., Culley E. J., Gladowski P., Goff V., Fong J., Marche S. M. (2010). Longitudinal analysis of the costs associated with inpatient initiation and subsequent outpatient continuation of proton pump inhibitor therapy for stress ulcer prophylaxis in a large managed care organization. *Journal of Managed Care Pharmacy*.

[8] Heidelbaugh J. J., Kim A. H., Chang R., Walker P. C. (2012). Overutilization of proton-pump inhibitors: what the clinician needs to know. *Therapeutic Advances in Gastroenterology*.

[9] Boath E. H., Blenkinsopp A. (1997). The rise and rise of proton pump inhibitor drugs: patients’ perspectives. *Social Science & Medicine*.

[10] Marks D. J. B. (2016). Time to halt the overprescribing of proton pump inhibitors. *Clinical Pharmacist*.

[11] Colombet I., Sabatier B., Gillaizeau F., Prognon P., Begue D., Durieux P. (2009). Long-term effects of a multifaceted intervention to encourage the choice of the oral route for proton pump inhibitors: an interrupted time-series analysis. *Quality and Safety in Health Care*.

[12] Skalli S., Bertin C., Charhon N. (2015). Pharmacist’s interventions on proton pump inhibitor prescriptions in a University Hospital. *Journal de Pharmacie de Belgique*.

[13] Valette S., Dory A., Gourieux B., Weber J. C. (2020). Evaluation of the implantation of a de-prescribing process for proton pump inhibitor (PPI) using an algorithm within an internal medicine department. *La Revue de Medecine Interne*.

[14] Thompson W., Hogel M., Li Y. (2016). Effect of a proton pump inhibitor deprescribing guideline on drug usage and costs in long-term care. *Journal of the American Medical Directors Association*.

[15] McDonald E. G., Jones J., Green L., Jayaraman D., Lee T. C. (2015). Reduction of inappropriate exit prescriptions for proton pump inhibitors: a before-after study using education paired with a web-based quality-improvement tool. *Journal of Hospital Medicine*.

[16] Hamzat H., Sun H., Ford J. C., MacLeod J., Soiza R. L., Mangoni A. A. (2012). Inappropriate prescribing of proton pump inhibitors in older patients. *Drugs & Aging*.

[17] Allenet B., Bedouch P., Rose F.-X. (2006). Validation of an instrument for the documentation of clinical pharmacists’ interventions. *Pharmacy World and Science*.

[18] Bedouch P., Charpiat B., Conort O. (2008). Assessment of clinical pharmacists’ interventions in French hospitals: results of a multicenter study. *The Annals of Pharmacotherapy*.

[19] Dooley M. J., Allen K. M., Doecke C. J. (2004). A prospective multicentre study of pharmacist initiated changes to drug therapy and patient management in acute care government funded hospitals. *British Journal of Clinical Pharmacology*.

[20] Bedouch P., Charpiat B., Roubille R. (2007). Site internet de la Société française de pharmacie clinique pour l’analyse des interventions pharmaceutiques: finalité, mode d’emploi et perspectives. *Journal de Pharmacie Clinique*.

[21] Alsultan M. S., Mayet A. Y., Malhani A. A., Alshaikh M. K. (2010). Pattern of intravenous proton pump inhibitors use in ICU and Non-ICU setting: a prospective observational study. *Saudi Journal of Gastroenterology: Official Journal of the Saudi Gastroenterology Association*.

[22] Dufay E., Morice S., Dony A. (2016). The clinical impact of medication reconciliation on admission to a French hospital: a prospective observational study. *European Journal of Hospital Pharmacy*.

[23] Greiver M., Dahrouge S., O’Brien P. (2019). Improving care for elderly patients living with polypharmacy: protocol for a pragmatic cluster randomized trial in community-based primary care practices in Canada. *Implementation Science*.

[24] Turner J. P., Richard C., Lussier M. T. (2018). Deprescribing conversations: a closer look at prescriber-patient communication. *Therapeutic Advances in Drug Safety*.

[25] Bedouch P., Sylvoz N., Charpiat B. (2015). Trends in pharmacists’ medication order review in French hospitals from 2006 to 2009: analysis of pharmacists’ interventions from the Act-IP website observatory. *Journal of Clinical Pharmacy and Therapeutics*.

[26] Ilickovic I., Jankovic S., Tomcuk A., Djedovic J. (2016 May). Pharmaceutical care in a long-stay psychiatric hospital. *European Journal of Hospital Pharmacy*.

[27] Heidelbaugh J. J., Metz D. C., Yang Y.-X. (2012). Proton pump inhibitors: are they overutilised in clinical practice and do they pose significant risk?. *International Journal of Clinical Practice*.

[28] Nguyen P. V.-Q., Tamaz R. (2018). Inappropriate prescription of proton pump inhibitors in a community setting. *Canadian Journal of Hospital Pharmacy*.

[29] Koczka C. P., Geraldino-Pardilla L. B., Goodman A. J. (2013). Physicians’ opinions of stress ulcer prophylaxis: survey results from a large urban medical center. *Digestive Diseases and Sciences*.

[30] Ghosh G., Schnoll-Sussman F., Mathews S., Katz P. O. (2020). Reported proton pump inhibitor side effects: what are physician and patient perspectives and behaviour patterns?. *Alimentary Pharmacology & Therapeutics*.

[31] Thompson W., Farrell B., Welch V. (2019). Continuation or deprescribing of proton pump inhibitors: a consult patient decision aid. *Canadian Pharmacists Journal/Revue des Pharmaciens du Canada*.

[32] Farrell B., Pottie K., Thompson W. (2017). Deprescribing proton pump inhibitors: evidence-based clinical practice guideline. *Canadian family physician Medecin de famille canadien*.

[33] Michelon H., Delahaye A., Fellous L. (2019). Proton pump inhibitors: why this gap between guidelines and prescribing practices in geriatrics?. *European Journal of Clinical Pharmacology*.

