# Supplementary material for: Characteristics of Pharmacists' Interventions Related to Proton-Pump Inhibitors in French Hospitals: An Observational Study
Source: Int J Clin Pract. 2022 Jun 28;2022:9619699. doi: 10.1155/2022/9619699 (PMC9256420; doi:10.1155/2022/9619699)
Supplement: Supplementary Materials — PPI-related DRPs among total DRPs according to year are presented in Appendix A. Nature of PPI-related DRPs according to year is presented in Appendix B. [file 9619699.f1.zip › 9619699.f1/Appendix A (1).docx]

**Appendix A: PPI-related DRPs amoung total DRPs according to year**

| **Year** | 2007 | 2008 | 2009 | 2010 | 2011 | 2012 | 2013 | 2014 | 2015 | 2016 | 2017 | 2018 | 2019 |
| --- | --- | --- | --- | --- | --- | --- | --- | --- | --- | --- | --- | --- | --- |
| **201Number of DRPs** | 12,958 | 21,482 | 21,072 | 25,823 | 31,569 | 34,550 | 38,100 | 60,493 | 55,933 | 72,933 | 77,431 | 83,723 | 84,553 |
| **Number of DRPs related to PPI** | 535 | 917 | 975 | 1,061 | 1,345 | 1,532 | 1,611 | 2,570 | 2,373 | 3,756 | 4,103 | 4,687 | 4,229 |
| **Percentage of DRP related to PPI (%)** | 4.13 | 4.27 | 4.63 | 4.11 | 4.26 | 4.43 | 4.23 | 4.25 | 4,24 | 5.60 | \|5.60 | 5.00 | 5.00 |
