# Supplementary material for: Characteristics of Pharmacists' Interventions Related to Proton-Pump Inhibitors in French Hospitals: An Observational Study
Source: Int J Clin Pract. 2022 Jun 28;2022:9619699. doi: 10.1155/2022/9619699 (PMC9256420; doi:10.1155/2022/9619699)
Supplement: Supplementary Materials — PPI-related DRPs among total DRPs according to year are presented in Appendix A. Nature of PPI-related DRPs according to year is presented in Appendix B. [file 9619699.f1.zip › 9619699.f1/Appendix B (1).docx]

**Appendix B: Nature of PPI-related DRPs according to year.**

|  | **Non-conformity to guidelines/**  **contraindication** | **Non-prescription of a drug available at hospital** | **Drug monitoring** | **Untreated indication** | **Subtherapeutic dosage** | **Supratherapeutic dosage** | **Drug use without indication** | **Drug interaction** | **Adverse drug reaction** | **Improper administration** | **Failure to receive drug** | ***P-value*** |
| --- | --- | --- | --- | --- | --- | --- | --- | --- | --- | --- | --- | --- |
| 2007 | 17 (3.2) | 161 (30.1) | 0 (0.0) | 5 (0.9) | 6 (1.1) | 105 (19.6) | 85 (15.9) | 15 (2.8) | 15 (2.8) | 125 (23.4) | 1 (0.2) | < 0.01^a^ |
| 2008 | 69 (7.5) | 296 (32.2) | 0 (0.0) | 17 (1.9) | 24 (2.6) | 91 (9.9) | 111 (12.1) | 80 (8.7) | 22 (2.4) | 205 (22.4) | 2 (0.3) |  |
| 2009 | 26 (2.7) | 341 (35.0) | 0 (0.0) | 10 (1.0) | 13 (1.3) | 111 (11.4) | 102 (10.5) | 204 (20.9) | 21 (2.2) | 144 (14.8) | 3 (0.2) |  |
| 2010 | 56 (5.3) | 226 (21.3) | 2 (0.2) | 6 (0.6) | 8 (0.8) | 162 (15.3) | 147 (13.9) | 250 (23.6) | 12 (1.1) | 187 (17.6) | 5 (0.5) |  |
| 2011 | 138 (13.0) | 277 (26.1) | 0 (0.0) | 15 (1.1) | 17 (1.3) | 258 (19.2) | 143 (10.6) | 190 (14.1) | 26 (1.9) | 268 (19.9) | 13 (1.0) |  |
| 2012 | 134 (8.7) | 264 (17.2) | 0 (0.0) | 27 (1.8) | 21 (1.4) | 241 (15.7) | 270 (17.6) | 148 (9.7) | 32 (2.1) | 379 (24.7) | 16 (1.0) |  |
| 2013 | 109 (6.8) | 353 (21.9) | 7 (0.4) | 46 (2.9) | 49 (3.0) | 292 (18.1) | 274 (17.0) | 120 (7.5) | 32 (2.0) | 318 (19.7) | 11 (0.7) |  |
| 2014 | 174 (6.8) | 703 (27.4) | 20 (0.8) | 101 (3.9) | 55 (2.1) | 423 (16.5) | 425 (16.5) | 203 (7.9) | 46 (1.8) | 404 (16.0) | 16 (0.6) |  |
| 2015 | 139 (5.9) | 547 (23.1) | 26 (1.1) | 103 (4.3) | 48 (2.0) | 467 (19.7) | 370 (15.6) | 122 (5.1) | 34 (1.4) | 493 (20.8) | 24 (1.0) |  |
| 2016 | 190 (5.1) | 1019 (27.1) | 13 (0.4) | 179 (4.8) | 126 (3.4) | 594 (15.8) | 777 (20.7) | 97 (2.6) | 31 (0.8) | 690 (18.4) | 40 (1.1) |  |
| 2017 | 151 (3.7) | 1242 (30.27) | 6 (0.2) | 268 (6.5) | 226 (5.5) | 622 (15.2) | 769 (18.7) | 97 (2.4) | 30 (0.7) | 660 (16.1) | 32 (0.8) |  |
| 2018 | 260 (5.6) | 1298 (27.7) | 11 (0.2) | 408 (8.7) | 209 (4.5) | 795 (17.0) | 974 (20.8) | 148 (3.2) | 34 (0.7) | 508 (10.8) | 42 (0.9) |  |
| 2019 | 294 (7.0) | 1023 (24.2) | 34 (0.8) | 426 (10.1) | 218 (5.2) | 629 (14.9) | 993 (23.5) | 103 (2.5) | 31 (0.7) | 471 (11.1) | 7 (0.2) |  |

^a^ Numbers of ’Drug monitoring’ and ‘Failure to receive drug’ DRPs were limited and were grouped for statistical analysis.
